# Two Cases Illustrating a Rapid Method of Healing Boil and Abscess Cavities

**Published:** 1900-04

**Authors:** M. B. Hutchins

**Affiliations:** Atlanta, Ga.


### TWO CASES ILLUSTRATING A RAPID METHOD OF HEALING BOIL AND ABSCESS CAVITIES.

BY M. B. HUTCHINS, M.D.,
ATLANTA, GA.

It is not customary to publish results in so few cases as named in the above title, but the method to be described has been used only in the two cases, and the effect of the treatment employed was so striking that I consider myself justified in bringing it to the attention of the profession, that it may receive wider employment.

The first case was that of a young lady who had an almost hen-egg sized subcutaneous abscess, following an infective folliculitis, in the right axilla. The patient being extremely nervous and sensitive to the slightest pain, I froze the skin over the abscess with ethyl chlorid and opened it without giving any pain. About one ounce of pus was discharged. The cavity having been quite well emptied, the usual packing was suggested, and declined on the plea of inability to endure the pain.

A poultice of flaxseed meal was then made with about three per cent. carbolized water, and applied directly over the wound and cavity, orders being given for frequent change of poultices. The next day the cavity was not only thoroughly emptied but the walls had begun to unite, and within three days the cavity was healed, the skin wound healing last, instead of first, as sometimes happens where the lips are not kept separate.

The second case was that of a neglected boil on the back of the neck, complete suppuration having occurred. The abscess was about the size of a walnut. It was freely opened and emptied and the flaxseed meal, carbolized poultice worn as in the other case. After two days there was nothing left but the slightest gaping incision, the cavity having disappeared.

The poultice made with carbolized water seems to owe its effectiveness to several things. Being applied directly over the mouth of the cavity, preferably with a layer of clean guaze or cloth intervening, it provides continuous drainage through absorption in the poultice, aided by the steady pressure. In this way all pus and necrotic tissue come away. The carbolic acid is sufficiently strong to inhibit the multiplication of pus organisms or continued reinfection. The pressure of the poultice also causes coaptation of the abscess walls, which seem, after being freed of dead tissue, to grow directly together. Meanwhile, the warmth and moisture of the poultice and the steady discharge prevent the often seen premature union of the cut edges, such as frequently happens even with pus beneath.

Packing the cavity with antiseptic gauze is a painful and troublesome procedure, reinfection is likely to take place in changing dressings, or if this does not happen the presence of the antiseptic in the cavity leads to the formation of a thin coagulum which interferes with healing.

The cases reported show the entire superiority of the antiseptic poultice method over any other.

In conclusion I may add that the use of only antiseptic poultices throughout in all cases of furuncle or abscess, where poultices are employed, will do much to prevent infection of adjacent areas with the possible final occurrence of general infection, as shown in crops of cutaneous lesions, or septicemia.

*305-306 Fitten Building.*

---

OPERATING with gloves has, we are glad to say, gone out of fashion in Germany. It never had any excuse except to save the surgeon from washing his hands thoroughly. Mickulicz, in Breslau, and Kocher, in Berne, still use gloves, although the latter is not so particular about them as he used to be. If any gloves are worn, the consensus of opinion seems to be that they should be made of rubber and not of cotton or silk. Every one who has used them, and discarded them, said that they interfered materially with the tactile sense, and the aseptic results were not improved sufficiently to make up for this disadvantage.—*Post-Graduate.*

